# Altered fractional amplitude of low frequency fluctuation associated with cognitive dysfunction in first-episode drug-naïve major depressive disorder patients

**DOI:** 10.1186/s12888-016-1190-1

**Published:** 2017-01-11

**Authors:** Manli Huang, Shaojia Lu, Liang Yu, Lingjiang Li, Peng Zhang, Jianbo Hu, Weihua Zhou, Shaohua Hu, Ning Wei, Jinwen Huang, Jian Weng, Yi Xu

**Affiliations:** 1 Department of Psychiatry, The First Affiliated Hospital, Zhejiang University School of Medicine, The Key Laboratory of Mental Disorder’s Management of Zhejiang Province, No. 79, Qingchun Road, Hangzhou, 310003 Zhejiang China; 2 Department of Anesthesiology and Pain, Hang Zhou First People’s Hospital, Hangzhou, Zhejiang China; 3 Mental Health Institute, The Second Xiangya Hospital, Central South University, Key Laboratory of Psychiatry and Mental Health of Hunan Province, National Technology Institute of Psychiatry and Mental Health, Changsha, Hunan China; 4 Mental Health Centre, Xiaoshan Hospital of Zhejiang Province, Hangzhou, Zhejiang China; 5 Bio-X Lab, Department of Physics, Zhejiang University, Hangzhou, Zhejiang China

**Keywords:** Major depressive disorder, Cognitive function, Fractional amplitude of low frequency fluctuation (fALFF)

## Abstract

**Background:**

Previous studies have demonstrated that abnormities of both resting-state brain activity and cognitive dysfunction are frequently observed in patients with major depressive disorder (MDD). However, the underlying relationship between these two aspects is less investigated. In this context, the aim of the present study was to investigate the association between cognitive dysfunction and altered resting-state brain function in first-episode drug-naïve MDD patients.

**Methods:**

Twenty-five drug-naïve MDD patients and twenty-six age-, sex-, and education-matched normal controls were recruited in this study. Cognitive function was evaluated by using a series of validated test procedures. The resting-state functional magnetic resonance imaging data were obtained on a Philips 3.0 Tesla scanner and analysed using the fractional amplitude of low frequency fluctuation (fALFF) method. Correlations of fALFF values with cognitive dysfunction were further analysed.

**Results:**

Compared with healthy controls, MDD patients showed significantly fewer completed categories in the Wisconsin Card Sorting Test (WCST) and decreased scores in the first and second subtests of the Continuous Performance Test (CPT). However, the two groups did not differ in their performance on the Stroop Colour Word Test and Trail-making Test. MDD patients exhibited significantly decreased fALFF values in the left superior frontal gyrus (SFG), left middle frontal gyrus, and left inferior frontal gyrus, as well as increased fALFF values in the left inferior temporal gyrus (ITG), bilateral parahippocampal gyrus, and the right caudate. Finally, the correlation analyses revealed that fALFF values in the left SFG and left ITG were associated with the number of WSCT completed categories and scores on the second subtest of the CPT in MDD, respectively.

**Conclusions:**

The present findings suggest that there is little evidence of an association between regional abnormalities in resting-state brain function and cognitive deficits in MDD.

## Background

Major depressive disorder (MDD) is one of the most common mental disorders and is characterized by persistent negative mood and altered cognitive functions [1]. MDD is estimated to threaten the health of 16% of the entire global population and contributes substantially to the global burden of disease and disability [2]. However, to date, the mechanisms underlying the development of depression remain unclear. In this context, plenty of studies have been directed to elucidate the pathophysiology of MDD, and MDD patients have been demonstrated to experience abnormalities of resting-state brain activity and deficits in cognitive function [3, 4].

In the last two decades, there have been a great amount of functional magnetic resonance imaging (fMRI) studies for MDD pointing to a role of functional brain impairments in the aetiology of the disease, and increasing evidence has accumulated for regional abnormalities within the limbic-cortical-striatal-pallidal-thalamic circuitry associated with MDD [5]. Resting-state fMRI is an effective way to characterize the resting-state brain activity without any tasks that may represent more than 95% of the total activity of the brain [6]. Recently, a high-quality meta-analysis summarized the previous findings of resting state fMRI studies in MDD, revealing a distributed pattern of brain regions with increased or decreased spontaneous activity in MDD. Decreased connectivity/activity in depression spatially converged mainly in the left superior/middle temporal gyrus and bilaterally in the insula, precuneus, superior frontal gyrus, lentiform nucleus and thalamus, while increased connectivity/activity was mainly found in the pre-/subgenual anterior cingulate cortex and neighbouring medial frontal cortex, the precuneus and neighbouring posterior cingulate cortex, bilaterally in the lateral prefrontal cortex with a left predominance, left lateral parietal cortex along with the right hippocampus and right cerebellum [7].

With regard to cognitive function, although not always consistent, findings for neuropsychological impairments such as in attention [8], memory [9], executive functioning [10], visuo-spatial processing and psychomotor function [11] in MDD patients have been observed in an emerging body of investigations [12]. Moreover, further studies have confirmed that cognitive dysfunction is observed not only in MDD patients with current mood episodes [13] but also in patients during remission of MDD [14]. Interestingly, a recent study by Vinberg et al. found that among healthy individuals with a heritable risk for affective disorder, onset of affective diseases in late life might be predicted by cognitive deficits, especially for executive function and attention [15]. Altogether, these findings indicate that cognitive dysfunction may exist in the early stages and subsequently persist into the whole course of MDD and, importantly, that cognitive dysfunction may play a role as a trait marker of MDD.

In addition to these findings, task-related fMRI studies have identified a number of functionally aberrant cortical and subcortical areas in patients with depression during paradigms examining cognitive functions such as attention, working memory and executive processing [16]. Performance in working memory tasks has been demonstrated to be associated with a dysfunctional activation of the medial orbitofrontal and rostral anterior cingulate cortex in MDD in a previous study [17]. Meanwhile, executive deficits in MDD have been repeatedly associated with a dysfunction of prefrontal cortical regions [18]. Taken together, all of these observations suggest that cognitive deficits might be associated with brain functional changes in MDD.

In a prior study, regional grey matter reduction has been proven to be related to cognitive deficits in MDD patients [19]. Conversely, although abnormities of both resting-state brain activity and cognitive dysfunction are frequently observed in patients with MDD, the potential relationship between these two aspects is unknown and less investigated. In this context, the aim of the present study was to investigate the association between cognitive dysfunction and altered resting-state brain function in drug-naïve MDD patients. To assess the regional spontaneous activity, Zang and colleagues proposed an amplitude of low-frequency fluctuation (ALFF) method [20]. Although the nature of the spontaneous low-frequency fluctuations (LFFs) is unclear, LFFs, which arise from neurovascular mechanisms [21], are believed to be associated with regional spontaneous neuronal activity [22]. However, ALFF is sensitive to the physiological noise; thus, Zou et al. proposed an improved ALFF method, namely, fractional ALFF (fALFF) approach to overcome this limitation [23]. The fALFF approach is an advanced technique to measure local fluctuations in neuronal activity, rather than generalized neuronal activity, which can provide a more specific index of low frequency oscillatory phenomena [24]. Compared to the traditional measurement of the ALFF, fALFF measurements have shown increased sensitivity and reduced bias from nonspecific physiological signal components [23]. The fALFF method has been widely used to probe the neural basis of individual differences in several mental disorders [25]. Motivated by previous work, we used fALFF in the current study.

## Method

### Participants

The study group comprised 51 subjects (male/female, 17/34), aged 18–44 years, including 25 drug-naïve patients with first-episode MDD (depression group) and 26 healthy controls (control group). MDD patients were recruited from the department of Psychiatry in The First Affiliated Hospital, Zhejiang University School of Medicine, Hangzhou, Zhejiang, P.R. China. The inclusion criteria were as follows: 1) met the Diagnostic and Statistical Manual of Mental Disorders, IV Edition (DSM-IV) criteria for current first episode unipolar MDD, which was assessed using the Structured Clinical Interview for DSM-IV (SCID) by two professional psychiatrists; 2) completed at least a junior middle school level of education; 3) had a score ≥ 17 on the 17-item Hamilton Depression Scale (HAMD) [26]; and 4) is of Han ethnicity and 5) right handed. Age- and sex-matched healthy volunteers were recruited from the local community and university via advertisements. The general exclusion criteria for all participants included were as follows: 1) treatment for depression; 2) significant medical illness; 3) a history of organic brain disease, cerebral trauma, seizure disorder, or MRI evidence of structural brain abnormalities; 4) the appearance of any other psychiatric axis-I or axis-II disorders (except MDD in patients) after SCID screening; 5) a family history of bipolar disorder; 6) a history of alcohol and drug abuse; 7) women pregnant, lactating or in a menstrual period, and 8) contraindications to MRI scan, including metallic implants, retractors or braces, and claustrophobia. Written informed consent was obtained, and this study was approved by the ethics committee of The First Affiliated Hospital, Zhejiang University School of Medicine.

### Procedures

#### Demographic and clinical assessments

The diagnoses were ascertained through the consensus of two board-certified psychiatrists on the basis of clinical interviews and administrations of the Structured Clinical Interview for DSM-IV (SCID). The demographic and clinical data were collected using a self-designed questionnaire for all participants. The severity of mood symptoms was assessed by using the 17-item HAMD [26], Hamilton Anxiety Rating Scale (HAMA) [27] and Montgomery Asberg Depression Rating Scale (MADRS) [28].

### Neuropsychological tests

A well-known neuropsychological test battery on attention, memory, processing speed, behavioural inhibition and executive function was administered to each subject, which included the Wisconsin Card Sorting Test (WCST) [29], Stroop Colour Word Test (SCWT) [30], Continuous Performance Test (CPT) [31], and Trail-making Test (TMT) [32]. All of these tests have been demonstrated to reveal cognitive deficits in MDD patients in numerous previous studies [19, 33].

1. Executive function was evaluated by using a computerized version of the WCST. This WCST comprised 48 cards and a maximum of 6 category switches. After 6 consecutive correct responses, subjects were asked to change the sorting principle to another category. The test ended when subjects completed all 6 categories correctly or used all 48 cards. We evaluated the primary efficacy outcome by using indices of the WCST for the performance of executive function that included total trials (TT), total correct (CT), total errors (TE), preservative errors (PE), random errors (RE), and categories completed.
2. The SCWT was conducted to measure selective attention/processing speed (SCWT A and SCWT B), behavioural inhibition (SCWT C), and executive function (SCWT interference). First, the subjects were asked to read out three written colours printed in black ink as fast as possible (SCWT A). Then, the subjects were instructed to name the colour as fast as possible (SCWT B). Finally, the subjects were required to name the ink colour of a colour word as fast as possible (SCWT C). The colour word was not the same as the ink colour. The performance for each condition was calculated by the processing time per item in seconds. The reaction time difference in part C relative to part B is called the ‘interference’ effect. Two subtasks were included: written colour name differing from printed colour ink matching and speaking.
3. The CPT was conducted to measure sustained and selective attention and impulsivity. It can be divided into 3 parts. In part 1, subjects were shown a series of numbers, and they were instructed to click the mouse whenever the number “4” was displayed. In part 2, eight numbers were shown, and they had to respond whenever a “4” was displayed. In part 3, eight numbers were shown, and they had to response whenever a “7” was displayed. The number of correct detections in all three parts and preservative errors in part 3 were measured.
4. The TMT, given in two parts, was administered to evaluate attentive and executive functioning. In TMT-A, the subject was required to quickly draw lines to connect consecutively numbered circles. In TMT-B, the subject was asked to alternately combine numbers with letters in ascending order. The task completion was measured in seconds. Part A measures visuo-spatial attention and performance speed, whereas Part B requires mental flexibility, ability to shift attention, and strategy.

### MRI acquisition

Magnetic resonance imaging was performed by experienced professional staff in The First Affiliated Hospital, Zhejiang University School of Medicine using the Philips Magnetic Resonance Imaging Systems Achieva 3.0 T TX (Philips Healthcare, Netherlands). An 8-channel SENSE head coil was used to emit and receive MRI signals. Head movement was reduced using foam pads, and earplugs were used to reduce noise stimulation. The subjects were instructed to relax, keep their eyes closed, stay awake, remain still, and not think of anything in particular. The subjects’ compliance was confirmed after the scanning was completed. First, a high-resolution T1-weighted sequence using a three-dimensional magnetization prepared rapid acquisition gradient echo sequence was used. Images of the whole brain were acquired in an axial orientation with the following parameters: repetition time (TR)/echo time (TE) = 7.5/3.7 ms, resolution = 240 × 227 matrix, slice thickness = 1 mm, 150 slices, field of view (FOV) = 240 mm × 240 mm, voxel size = 1 × 1 × 1 mm^3^, flip angle = 8°, and scan time = 4′15″. The functional images were recorded axially over 6′48″ using a field echo-planar imaging (EPI) sequence with the following parameters: TR/TE = 2000/35 ms, flip angle = 80°, 24 slices, slice thickness/gap = 5.0/1.0 mm, voxel size = 2.4 × 2.4 × 5.0 mm^3^, resolution = 100 × 100 matrix, FOV = 240 mm × 240 mm.

#### Analysis of MRI data

The first 10 functional volumes were discarded for signal equilibrium and patient adaptation to the scanning noise. Image preprocessing was performed using the Data Processing Assistant for RS-fMRI (DPARSF) version 2.0 software package (http://restfmri.net/forum/DPARSF) [34], which was a convenient plug-in software in the Statistical Parametric Mapping 8 (SPM8, http://www.fil.ion.ucl.ac.uk/spm) and RS-fMRI Data Analysis Toolkit (REST, http://www.restfmri.net) [35]. Functional data preprocessing included slice timing correction, motion correction, and spatial normalization. Head motion of more than either 2.0 mm maximum displacement in any of the x, y, or z directions or 2.5° in any angular motion throughout the course of the scan was found in any participant. The normalized functional data underwent spatial smoothing [4-mm full width at half-maximum (FWHM) Gaussian kernel] and removed the linear trend of time courses, as previously described [21, 36]. ALFF values were calculated using DPARSF software. The time series for each voxel was first transformed to the frequency domain using a fast Fourier transform (FFT), and then the sum of the frequencies in the low frequency band (0.01–0.08Hz) was computed. Quadratic detrending was not performed as the removal of filters below 0.01Hz was a non-linear detrending. The ALFF measure at each voxel was the averaged square root of the power in the 0.01–0.08Hz window, normalized by the mean within-brain ALFF value for that subject. This averaged square root was taken as the amplitude of LFF (ALFF, Eq. 1) [20]. The fALFF value was the ratio of the power spectrum of low-frequency (0.01–0.08Hz) to that of the entire frequency range. In fact, as shown in Eq. 2, fALFF can be regarded as a normalized ALFF, using the total energy over the detectable frequency range [37].

1 $$ ALFF={\displaystyle {\sum}_{k:{f}_k\in \left[0.01,0.08\right]}\sqrt{\frac{a_k^2(f)+{b}_k^2(f)}{N}}} $$

2 $$ fALFF=\raisebox{1ex}{${\displaystyle {\sum}_{k:{f}_k\in \left[0.01,0.08\right]}}\sqrt{\frac{a_k^2(f)+{b}_k^2(f)}{N}}$}\!\left/ \!\raisebox{-1ex}{${\displaystyle {\sum}_{k=1}^N}\sqrt{\frac{a_k^2(f)+{b}_k^2(f)}{N}}$}\right. $$

### Statistical analysis

Between-group differences in demographic variables were examined using independent two-sample *t* tests for continuous variables and chi-square tests for categorical variables using the Statistical Package for the Social Sciences (SPSS) version 18.0 (IBM, Chicago, IL, USA). The level of two-tailed statistical significance was set at *p* < 0.05 for all tests.

To explore the fALFF differences between two groups, a second-level random-effect two-sample *t* test was performed in a voxel-by-voxel manner. Significant differences were set at a corrected significance level of *p* < 0.05 [combined height threshold *p* < 0.01 (*T* > 2.40) and a minimum cluster size of 18 voxels]. This correction was made using the AlphaSim program in the REST software (http://restfmri.net/forum/rest) with the parameter FWHM = 4 mm. This program applied the Monte Carlo simulation using both the individual voxel probability threshold and the cluster size to calculate the probability of detecting a false positive [35].

Furthermore, to evaluate any correlations between fALFF values and cognitive tests, whole brain multiple regression analyses integrated in SPM basic models were performed, and the threshold was set at *p* < 0.05, AlphaSim corrected. Then, the mean fALFF values of the surviving clusters were extracted by using region of interest (ROI) analyses. Correlations were conducted using Pearson’s product moment.

## Result

### Demographic, clinical, and neuropsychological testing results

As indicated in Table 1, the two groups did not significantly differ in age, gender, and education level. For the WSCT, patients with MDD completed significantly fewer categories than the healthy controls. In the CPT tests, MDD patients showed lower scores in the first and second subtests. However, there was no significant difference between the two groups in the SCWT and TMT. For more details, please refer to Table 2.

**Table 1:** Demographic and clinical characteristics for all subjects (mean ± SD)

	Depression group (*n* = 25)	Control group (*n* = 26)	*p* value
Age (years)	31.4 ± 7.26	28.7 ± 7.61	0.200
Gender (M/F)	7/18	10/16	0.428
Education (years)	12.0 ± 3.09	12.5 ± 3.09	0.613
Disease duration (months)	12.1 ± 10.4		
HAMD	25.6 ± 5.72		
MADRS	31.8 ± 7.76		
HAMA	25.9 ± 8.27		

*HAMD* Hamilton Depression Rating Scale, *MADRS* Montgomery-Asberg Depression Rating Scale, *HAMA* Hamilton Anxiety Rating Scale

**Table 2:** Comparisons of neuropsychological scores (Mean ± SD)

		Depression group (*n* =25)	Control group (*n* = 26)	*p* value
WSCT	TT	46.0 ± 6.50	46.8 ± 2.19	0.552
	CT	28.7 ± 9.67	33.0 ± 7.55	0.172
	TE	17.3 ± 8.57	14.7 ± 8.71	0.276
	PE	11.6 ± 8.13	9.15 ± 6.09	0.229
	RE	5.48 ± 2.82	5.50 ± 3.30	0.982
	Categories	3.52 ± 1.94	4.58 ± 1.68	0.042
SCWT	SCWT A	47.4 ± 17.8	48.0 ± 24.4	0.916
	SCWT B	77.4 ± 31.3	63.6 ± 21.3	0.070
	SCWT C	117.8 ± 37.9	103.4 ± 23.7	0.108
	SCWT interference	40.3 ± 17.3	39.8 ± 16.4	0.920
CPT	CPT 1	9.36 ± 2.66	10.7 ± 0.75	0.021
	CPT 2	9.12 ± 3.42	11.1 ± 1.86	0.012
	CPT 3	10.8 ± 2.44	11.5 ± 1.73	0.193
	CPT PE	0.60 ± 1.26	1.04 ± 1.80	0.320
TMT (s)	TMT A	63.4 ± 37.4	57.3 ± 40.8	0.583
	TMT B	117.3 ± 61.6	97.7 ± 77.4	0.324

*WSCT* Wisconsin Card Sorting Test, *TT* number of total trials, *CT* number of correct trials, *TE* total number of errors, *PE* preservative errors, *RE* random errors, *SWCT* Stroop Color Word Test, *CPT* Continuous Performance Test, *TMT* Trail-making test

### Neuroimaging with fALFF

**Fig. 1:**
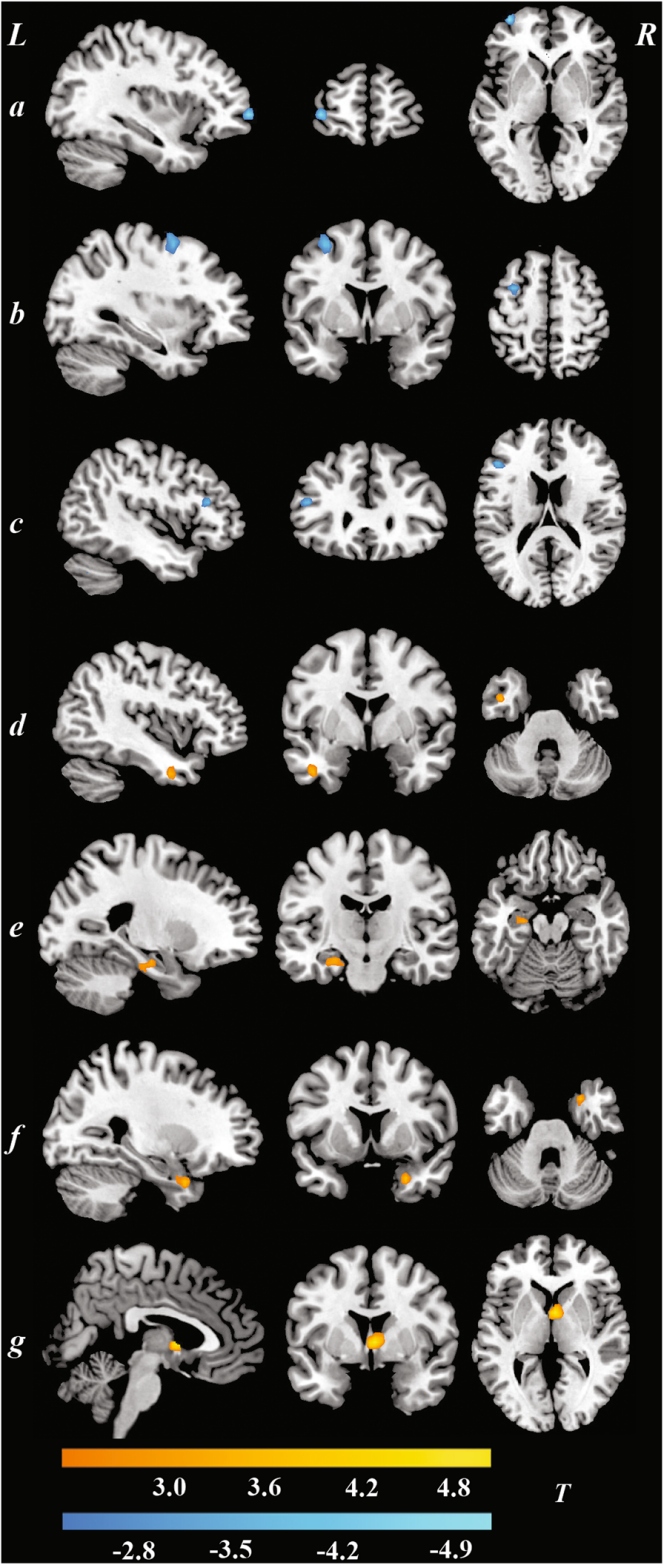
Brain regions showed decreased/increased fALFF values in depression patients as compared with normal controls (*p* < 0.05, AlphaSim corrected). **a** Superior frontal gyrus; **b** Middle frontal gyrus; **c** Inferior frontal gyrus; **d** Inferior temporal gyrus; **e** left Parahippocampal gyrus; **f** right Parahippocampal gyrus; **g** Caudate

MDD patients showed significantly lower fALFF values in the left superior frontal gyrus, left middle frontal gyrus, and left inferior frontal gyrus than that observed in the control group. Meanwhile, patients with MDD also showed increased fALFF values in the left inferior temporal gyrus, bilateral parahippocampal gyrus, and the right caudate. (see Fig. 1 and Table 3).

**Table 3:** Brain regions showing altered fALFF values in depression patients as compared with normal controls (*p* < 0.05, AlphaSim corrected)

Brain region	Hemisphere	Cluster size	*T* value	MNI coordinate		
				*x*	*y*	*z*
Decreased fALFF						
Superior frontal gyrus	L	19	−5.06	−36	60	0
Middle frontal gyrus	L	29	−3.94	−33	3	57
Inferior frontal gyrus	L	28	−3.82	−45	30	18
Increased fALFF						
Inferior temporal gyrus	L	33	3.59	−42	0	−33
Parahippocampal gyrus	L	42	3.33	−24	−15	−18
	R	19	3.77	27	9	−30
Caudate	R	29	4.92	3	3	3

*MNI* Montreal Neurological Institute

### Correlations

**Fig. 2:**
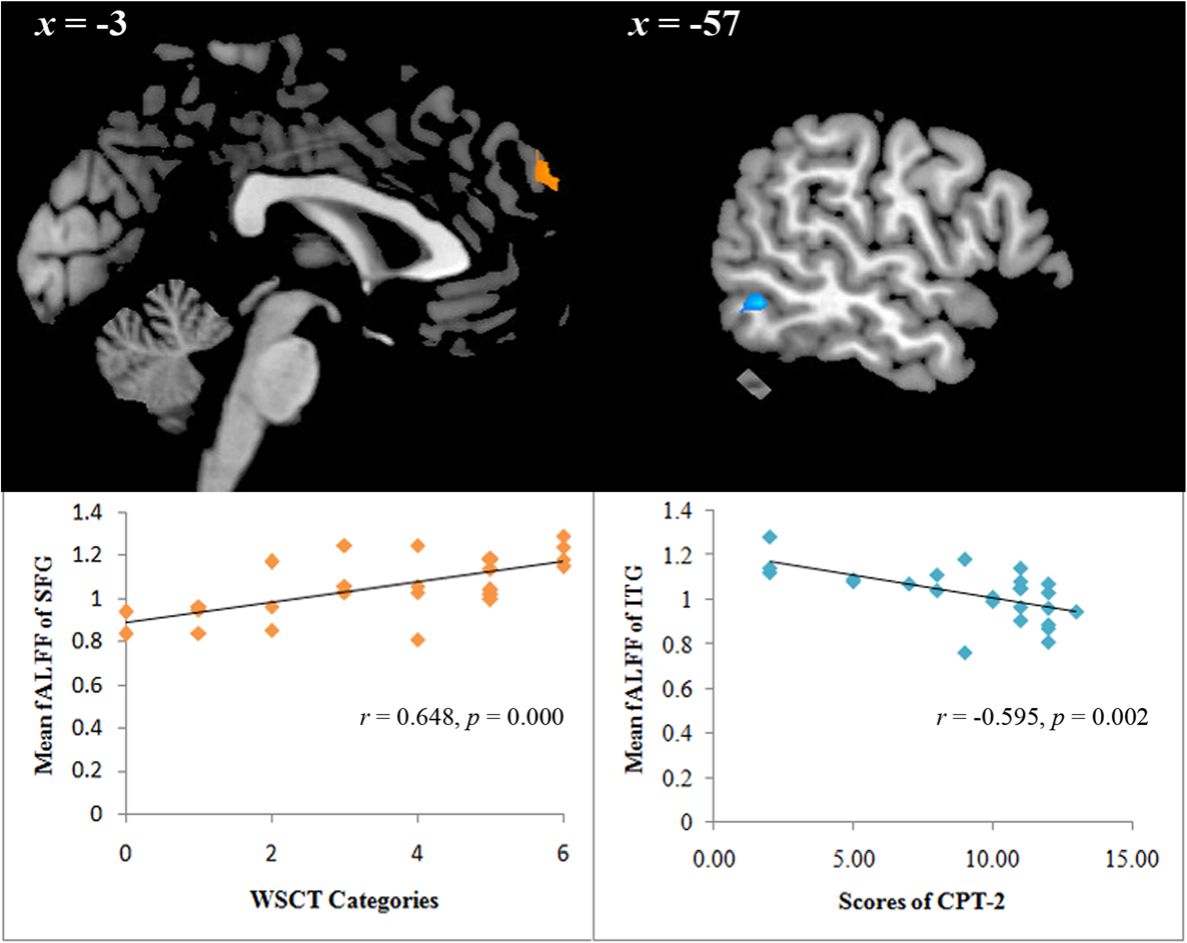
Correlation analysis between cognitive deficits and fALFF values in depression patients (*p* < 0.05, corrected). SFG, Superior frontal gyrus; ITG, Inferior temporal gyrus

In MDD patients, the whole-brain linear regression analyses conducted with SPM8 revealed that fALFF values in the left superior frontal gyrus (*x* = -3, *y* = 57, *z* = 36, cluster = 23, *T* = 3.63) and left inferior temporal gyrus (*x* = -57, *y* = -54, *z* = -9, cluster = 22, *T* = -3.87) were associated with the number of WSCT completed categories and CPT-2 scores, respectively. (see Fig. 2). These associations were not found in healthy controls.

## Discussion

In this study, cognitive function and resting-state brain function as revealed by fALFF were tested in first-episode drug-naïve MDD patients. The current findings suggested that MDD patients exhibited cognitive dysfunction in many aspects, including executive function, sustained and selective attention, and impulsivity. Meanwhile, this study also demonstrated that extensive regions involved in mood regulation and cognitive function showed altered resting-state brain function in MDD patients. Moreover, the correlation analyses revealed that cognitive deficits might be associated with regional abnormalities in resting-state brain function. To our knowledge, the present results are consistent with currently available functional neuroimaging and neuropsychological findings in MDD [13, 38].

Cognitive dysfunction in MDD patients has been commonly reported in prior studies. A meta-analysis of all studies published between 1975 and 1997 indicated that individuals with major depression were shown to have a consistent global-diffuse impairment in brain functions, with particular involvement of the frontal lobes, as characterized by executive dysfunction [39]. In another meta-analysis, McDermott et al. further reported significant correlations between depression severity and executive dysfunction; moreover, they demonstrated that there were positive associations between depression severity and cognitive deficits in the domains of episodic memory and processing speed as well [40]. Together with our results, these findings indicate that several aspects of cognitive impairment exist in MDD patients. However, the present study did not find any group differences in the SCWT and TMT. This might result from a heterogeneity of research samples since the mean disease course of MDD patients was relatively short and all of the patients in our study sample were experiencing their first episode of depression. Furthermore, the relatively high educational level in our sample could be another influential factor. People with higher educational levels have been reported to have greater cognitive reserve than those with lower educational levels, and this cognitive reserve might enable individuals to sustain brain damage and maintain function [41].

In the present study, significant functional abnormalities in brain regions involved in mood regulation and cognitive function [42] were identified in MDD patients, which was generally consistent with most previous findings. Our data were, at least in part, comparable with findings of a prior resting-state fMRI study which demonstrated that depression patients showed higher fALFF values in the left temporal subgyral region and lower fALFF values in the right frontal subcallosal gyrus. Interestingly, this study found an association between severity of depressive symptoms and altered resting-state brain function in depression patients as well [43]. In addition, previous resting-state fMRI studies using the fALFF method also reported that MDD patients showed altered brain function in widespread regions, including the parahippocampal gyrus, middle frontal gyrus, median frontal gyrus, and inferior temporal gyrus [44–47], which was comparable with our findings.

Moreover, the current study also explored the relationship between these MDD-associated brain functional abnormalities and cognitive deficits, potentially linking resting-state brain dysfunction with behavioural manifestations of MDD. In a recent study, regional grey matter reduction has been proven to be related with cognitive deficits in MDD patients. Impaired WCST performance was associated with decreased grey matter concentration in the left inferior temporal cortex, right orbitofrontal cortex, and right dorsolateral prefrontal cortex [19]. In fMRI studies, working memory and executive deficits in MDD have also been demonstrated to be associated with dysfunction of prefrontal cortical regions [48]. In the present study, we found that executive dysfunction was associated with altered activity of the left superior frontal gyrus in MDD patients but not healthy controls. The superior frontal gyrus is involved in cognitive processes, such as executive function and memory retrieval [49], which may help to explain the current association found in MDD patients. Furthermore, a negative association between the CPT-2 scores and fALFF values in the left inferior temporal gyrus was observed in MDD patients as well. In a previous study, it was reported that decreased metabolism of the inferior temporal gyrus was correlated with poor performance in the CPT in schizophrenia patients [50], which suggested that the attention deficit might be due to dysfunction of the inferior temporal gyrus, but it is still unclear how this region is related to attentional function. Taken together, these findings may help to provide evidence for elucidating the potential brain mechanisms underlying cognitive impairments in MDD.

## Limitations

Some limitations must be considered when interpreting our findings. First, the size of the study sample could be an issue. Although it was suitable for fALFF analysis, the sample size in each group was relatively small, which might result in some subtle functional changes in the brain not being observed [51]. In addition, the inclusion criteria for education level in this study might be a possible bias and confounding issue. Furthermore, the slice thickness and the parameter of the EPI sequence may have limited the resolution and accuracy of the fALFF results. Additionally, the present study was cross-section designed, restricting causal analysis. Finally, in the current analyses, only changes in regional connectivity could be detected, and it was not possible to determine how MDD affected the interactions among brain network systems.

## Conclusions

In conclusion, the present findings suggest that there is little evidence of an association between regional abnormalities in resting-state brain function and cognitive deficits in MDD.

## References

[1] Lu S, Peng H, Wang L, Vasish S, Zhang Y, Gao W, Wu W, Liao M, Wang M, Tang H (2013). Elevated specific peripheral cytokines found in major depressive disorder patients with childhood trauma exposure: a cytokine antibody array analysis. Compr Psychiatry.

[2] Kessler RC, Berglund P, Demler O, Jin R, Koretz D, Merikangas KR, Rush AJ, Walters EE, Wang PS, National Comorbidity Survey R (2003). The epidemiology of major depressive disorder: results from the National Comorbidity Survey Replication (NCS-R). Jama.

[3] Zhang X, Zhu X, Wang X, Zhong M, Yi J, Rao H, Yao S (2014). First-episode medication-naive major depressive disorder is associated with altered resting brain function in the affective network. PLoS One.

[4] Baune BT, Fuhr M, Air T, Hering C (2014). Neuropsychological functioning in adolescents and young adults with major depressive disorder--a review. Psychiatry Res.

[5] Sheline YI (2000). 3D MRI studies of neuroanatomic changes in unipolar major depression: the role of stress and medical comorbidity. Biol Psychiatry.

[6] Gao W, Jiao Q, Lu S, Zhong Y, Qi R, Lu D, Xiao Q, Yang F, Lu G, Su L (2014). Alterations of regional homogeneity in pediatric bipolar depression: a resting-state fMRI study. BMC psychiatry.

[7] Sundermann B, Olde Lutke Beverborg M, Pfleiderer B (2014). Toward literature-based feature selection for diagnostic classification: a meta-analysis of resting-state fMRI in depression. Frontiers in human neuroscience.

[8] Ravnkilde B, Videbech P, Clemmensen K, Egander A, Rasmussen NA, Rosenberg R (2002). Cognitive deficits in major depression. Scand J Psychol.

[9] Harmer CJ, Clark L, Grayson L, Goodwin GM (2002). Sustained attention deficit in bipolar disorder is not a working memory impairment in disguise. Neuropsychologia.

[10] Paelecke-Habermann Y, Pohl J, Leplow B (2005). Attention and executive functions in remitted major depression patients. J Affect Disord.

[11] Mondal S, Sharma VK, Das S, Goswami U, Gandhi A (2007). Neuro-cognitive functions in patients of major depression. Indian J Physiol Pharmacol.

[12] Baune BT, Miller R, McAfoose J, Johnson M, Quirk F, Mitchell D (2010). The role of cognitive impairment in general functioning in major depression. Psychiatry Res.

[13] Harvey PO, Le Bastard G, Pochon JB, Levy R, Allilaire JF, Dubois B, Fossati P (2004). Executive functions and updating of the contents of working memory in unipolar depression. J Psychiatr Res.

[14] Hammar A, Ardal G (2009). Cognitive functioning in major depression--a summary. Front Hum Neurosci.

[15] Vinberg M, Miskowiak KW, Kessing LV (2013). Impairment of executive function and attention predicts onset of affective disorder in healthy high-risk twins. J Clin Psychiatry.

[16] Mayberg HS (2003). Modulating dysfunctional limbic-cortical circuits in depression: towards development of brain-based algorithms for diagnosis and optimised treatment. Br Med Bull.

[17] Rose EJ, Simonotto E, Ebmeier KP (2006). Limbic over-activity in depression during preserved performance on the n-back task. NeuroImage.

[18] Wang L, LaBar KS, Smoski M, Rosenthal MZ, Dolcos F, Lynch TR, Krishnan RR, McCarthy G (2008). Prefrontal mechanisms for executive control over emotional distraction are altered in major depression. Psychiatry Res.

[19] Vasic N, Walter H, Hose A, Wolf RC (2008). Gray matter reduction associated with psychopathology and cognitive dysfunction in unipolar depression: a voxel–based morphometry study. J Affect Disord.

[20] Zang YF, He Y, Zhu CZ, Cao QJ, Sui MQ, Liang M, Tian LX, Jiang TZ, Wang YF (2007). Altered baseline brain activity in children with ADHD revealed by resting-state functional MRI. Brain Dev.

[21] Biswal B, Yetkin FZ, Haughton VM, Hyde JS (1995). Functional connectivity in the motor cortex of resting human brain using echo-planar MRI. Magn Reson Med.

[22] Fox MD, Raichle ME (2007). Spontaneous fluctuations in brain activity observed with functional magnetic resonance imaging. Nat Rev Neurosci.

[23] Zou QH, Zhu CZ, Yang Y, Zuo XN, Long XY, Cao QJ, Wang YF, Zang YF (2008). An improved approach to detection of amplitude of low-frequency fluctuation (ALFF) for resting-state fMRI: fractional ALFF. J Neurosci Methods.

[24] Liu CH, Ma X, Wu X, Fan TT, Zhang Y, Zhou FC, Li LJ, Li F, Tie CL, Li SF (2013). Resting-state brain activity in major depressive disorder patients and their siblings. J Affect Disord.

[25] Hoptman MJ, Zuo XN, Butler PD, Javitt DC, D'Angelo D, Mauro CJ, Milham MP (2010). Amplitude of low-frequency oscillations in schizophrenia: a resting state fMRI study. Schizophr Res.

[26] Hamilton M (1967). Development of a rating scale for primary depressive illness. Br J Soc Clin Psychol.

[27] Hamilton M (1959). The assessment of anxiety states by rating. Br J Med Psychol.

[28] Williams JB, Kobak KA (2008). Development and reliability of a structured interview guide for the Montgomery Asberg Depression Rating Scale (SIGMA). Br J Psychiatry.

[29] Monchi O, Petrides M, Petre V, Worsley K, Dagher A (2001). Wisconsin card sorting revisited: distinct neural circuits participating in different stages of the task identified by event-related functional magnetic resonance imaging. J Neurosci.

[30] Howieson DB, Lezak MD, Loring DW. Orientation and attention. Neuropsychological assessment. New York: Oxford University Press; 2004.

[31] Beck LH, Bransome ED, Mirsky AF, Rosvold HE, Sarason I (1956). A continuous performance test of brain damage. J Consult Psychol.

[32] Arnett JA, Labovitz SS (1995). Effect of physical layout in performance of the trail making test. Psychol Assess.

[33] Roca M, Lopez-Navarro E, Monzon S, Vives M, Garcia-Toro M, Garcia-Campayo J, Harrison J, Gili M (2015). Cognitive impairment in remitted and non-remitted depressive patients: a follow-up comparison between first and recurrent episodes. Eur Neuropsychopharmacol.

[34] Chao-Gan Y, Yu-Feng Z (2010). DPARSF: A MATLAB toolbox for “pipeline” data analysis of resting-state fMRI. Front Syst Neurosci.

[35] Song XW, Dong ZY, Long XY, Li SF, Zuo XN, Zhu CZ, He Y, Yan CG, Zang YF (2011). REST: a toolkit for resting-state functional magnetic resonance imaging data processing. PLoS One.

[36] Lowe MJ, Mock BJ, Sorenson JA (1998). Functional connectivity in single and multislice echoplanar imaging using resting-state fluctuations. Neuroimage.

[37] Zuo XN, Di Martino A, Kelly C, Shehzad ZE, Gee DG, Klein DF, Castellanos FX, Biswal BB, Milham MP (2010). The oscillating brain: complex and reliable. NeuroImage.

[38] Kerestes R, Davey CG, Stephanou K, Whittle S, Harrison BJ (2014). Functional brain imaging studies of youth depression: a systematic review. Neuroimage Clin.

[39] Veiel HO (1997). A preliminary profile of neuropsychological deficits associated with major depression. J Clin Exp Neuropsychol.

[40] McDermott LM, Ebmeier KP (2009). A meta-analysis of depression severity and cognitive function. J Affect Disord.

[41] Zhang S, Ou R, Chen X, Yang J, Zhao B, Yuan X, Wei Q, Cao B, Shang HF (2016). Correlative factors of cognitive dysfunction in PD patients: a cross-sectional study from Southwest China. Neurol Res.

[42] Disner SG, Beevers CG, Haigh EA, Beck AT (2011). Neural mechanisms of the cognitive model of depression. Nat Rev Neurosci.

[43] Lai CH, Wu YT (2015). The patterns of fractional amplitude of low-frequency fluctuations in depression patients: the dissociation between temporal regions and fronto-parietal regions. J Affect Disord.

[44] Liu F, Guo W, Liu L, Long Z, Ma C, Xue Z, Wang Y, Li J, Hu M, Zhang J (2013). Abnormal amplitude low-frequency oscillations in medication-naive, first-episode patients with major depressive disorder: a resting-state fMRI study. J Affect Disord.

[45] Wang L, Dai W, Su Y, Wang G, Tan Y, Jin Z, Zeng Y, Yu X, Chen W, Wang X (2012). Amplitude of low-frequency oscillations in first-episode, treatment-naive patients with major depressive disorder: a resting-state functional MRI study. PloS one.

[46] Jing B, Liu CH, Ma X, Yan HG, Zhuo ZZ, Zhang Y, Wang SH, Li HY, Wang CY (2013). Difference in amplitude of low-frequency fluctuation between currently depressed and remitted females with major depressive disorder. Brain Res.

[47] Tadayonnejad R, Yang S, Kumar A, Ajilore O (2014). Clinical, cognitive, and functional connectivity correlations of resting-state intrinsic brain activity alterations in unmedicated depression. J Affect Disord.

[48] Vasic N, Wolf RC, Walter H (2007). Executive functions in patients with depression. The role of prefrontal activation. Der Nervenarzt.

[49] Koechlin E, Hyafil A (2007). Anterior prefrontal function and the limits of human decision-making. Science.

[50] Siegel BV, Nuechterlein KH, Abel L, Wu JC, Buchsbaum MS (1995). Glucose metabolic correlates of continuous performance test performance in adults with a history of infantile autism, schizophrenics, and controls. Schizophr Res.

[51] Wei X, Shen H, Ren J, Li X, Xu X, Yang R, Lai L, Chen L, Hu J, Liu W (2014). Altered resting-state connectivity in college students with nonclinical depressive symptoms. PloS one.

